# Targeting oncogenic SOX2 in human cancer cells: therapeutic application

**DOI:** 10.1007/s13238-019-00673-x

**Published:** 2019-11-20

**Authors:** Shizhen Zhang, Yi Sun

**Affiliations:** 1 Division of Radiation and Cancer Biology, Department of Radiation Oncology, University of Michigan, Ann Arbor, MI 48109 USA; 2 The Cancer Institute of the Second Affiliated Hospital and Institute of Translational Medicine, Zhejiang University School of Medicine, Hangzhou, 310029 China

Sry-related high-mobility box 2 (SOX2) is a critical transcription factor that plays an important role in various phases of embryonic development and maintenance of undifferentiated embryonic stem cells (ESCs) (Feng and Wen, 2015). SOX2, as one of the Yamanaka factors, is involved in the conversion of mouse embryonic fibroblast cells (MEFs) into induced pluripotent stem cells (iPSCs) (Takahashi and Yamanaka, 2006). Recently, *SOX2* amplification, usually couples with aberrantly increased expression, were found in various human cancers, including breast, lung, esophagus, colon, prostate, ovarian among the others (Novak et al., 2019). SOX2 overexpression promotes cancer progression by accelerating cell proliferation, colony formation, migration, invasion, and sphere formation. Furthermore, SOX2 is causally related to the development of the resistance of cancer cells to chemotherapy, radiotherapy and targeted therapy in different types of human cancers, likely due to its ability to maintain the stemness of cancer stem cells (CSCs), which are defined as a subpopulation within tumor cells being equipped with stem cell-like properties that survives the treatment and initiates tumor progression (Novak et al., 2019). Thus, SOX2 has been validated as an attractive anti-cancer target (Huser et al., 2018).

Given its biological significance, SOX2 levels are precisely controlled by a complicated network of transcriptional, post-transcriptional, and post-translational regulators. At the transcriptional level, the two isoforms of E2f transcription factor 3 (E2f3), E2f3a and E2f3b, regulate SOX2 expression in a reciprocal way (Wuebben and Rizzino, 2017). The cyclin-dependent kinase inhibitor p21 directly binds to the SOX2 enhancer and negatively regulates SOX2 transcription (Wuebben and Rizzino, 2017), whereas transforming growth factor-β (TGFβ) or sirtuin 1 (SIRT1) induces SOX2 via SOX4 (Wuebben and Rizzino, 2017), or via chromatin-based epigenetic modification (Liu et al., 2016), respectively. More recently, a homeobox-containing transcription factor, muscle segment homeobox-2 (MSX2) was shown to destabilize the pluripotency circuitry by direct binding to the SOX2 promoter to repress SOX2 transcription (Wu et al., 2015). At the post-transcriptional level, SOX2 is subjected to regulation by several microRNAs and long non-coding RNAs in cancer cells (Wuebben and Rizzino, 2017). Finally, at the post-translational level, SOX2 protein is modulated by phosphorylation, sumoylation, methylation, acetylation and ubiquitylation. Phosphorylation of SOX2 at S249, S250 and S251 residues affects its stability (Wuebben and Rizzino, 2017), whereas sumoylation of SOX2 impairs its DNA-binding property and thus inhibiting its transcription activity (Wuebben and Rizzino, 2017). Notably, SOX2 is subjected to ubiquitylation by ubiquitin-conjugating enzyme UBE2S and CUL4A^DET1-COP1^ E3 ligase, respectively (Cui et al., 2018; Wuebben and Rizzino, 2017). The methylation of SOX2 is required for ubiquitylation and proteolysis mediated by HECT domain-containing WWP2 E3 ligase (Wuebben and Rizzino, 2017), and the CRL4^DCAF5^ ubiquitin ligase complex (Zhang et al., 2019).

Although SOX2 is highly relevant to cancer initiation, progression and development of drug resistance, directly targeting SOX2 has been proved to be difficult, since SOX2 is an “undrugable” transcription factor. The multiple preclinical studies have shown that SOX2 knockdown mediated by siRNAs, shRNAs or miRNAs dramatically suppresses proliferation and invasion of cancer cells in both *in vitro* cell culture and *in vivo* xenograft tumor models (Huser et al., 2018). These studies validate SOX2 as a promising target, but offer little therapeutic value due to huge challenge in efficacy and delivery. On the other hand, zinc-finger (ZF)-based artificial transcription factors (ATFs), which bind genomic sequences with potentially single locus specificity, provide an opportunity to modify, edit, and sculpt the epigenetic and transcriptional state of endogenous promoters. Indeed, ATFs that bind to the proximal *SOX2* promoters were shown to cause ~95% reduction of endogenous *SOX2* mRNA and protein in breast cancer cells upon retrovirus-based delivery (Huser et al., 2018). Significantly, these ATFs efficiently inhibited tumor growth in a xenograft model of breast cancer with the inhibitory effect maintained for a long term (Huser et al., 2018). Again, the challenge in the *in vivo* delivery of ATFs to solid tumor tissues constrains the clinical applications.

Given these limitations, targeting signal molecules upstream or downstream of SOX2 become attractive alternative approaches. Several small molecules have been reported to down-regulate SOX2 expression. For examples, a small molecular inhibitor of LSD1 (CBB1007) significantly reduced SOX2 expression and suppressed growth of tumor cells. Mechanistically, LSD1 inactivation enhanced repressive H3K9 methylations on the *Sox2* gene (Zhang et al., 2013). This newly developed inhibitor, however, is still in a preclinical stage and its potential clinical application is unknown. Furthermore, the cationic triphenylmethane pharmacophore gentian violet (GV) has been recently reported to suppress survival and self-renewal of melanoma cells through the inhibition of SOX2. Mechanistic study revealed that GV strongly decreased signal transducer and activator of transcription 3 (STAT3) phosphorylation at Tyr^705^ through an epidermal growth factor receptor (EGFR)-dependent manner, leading to reduced STAT3 nuclear translocation for *SOX2* promoter binding (Pietrobono et al., 2016). Given that GV is initially used as an antimycotic and antibacterial agent, its clinical application as an anticancer agent awaits further investigation. Finally, EGFR inhibitors, Gefitinib and Erlotinib, and Src inhibitor, Dasatinib, are all shown to reduce the levels of SOX2 by blocking the EGFR/SRC/AKT signaling, eventually suppressing the self-renewal properties of cancer stem cells in non-small cell lung cancer (Singh et al., 2012). Although various EGFR inhibitors are currently under clinical use, it is unknown how much its anti-cancer activity is attributable to SOX2 depletion.

Most recently, we found that neddylation inhibitor, MLN4924, also known as Pevonedistat, a small molecule currently in phase II clinical trials for anticancer application (Zhou et al., 2018), effectively depletes SOX2 via targeting the FBXW2-MSX2 axis (Yin et al., 2019). Neddylation is a reversible process catalyzed in a three-step enzymatic cascade by E1 NEDD8-activating enzyme (NAE), E2 NEDD8-conjugating enzymes (UBE2M/UBC12 or UBE2F), and NEDD8 E3 ligases, leading to attachment of ubiquitin-like NEDD8 to a lysine residue of targeted substrates (Zhao et al., 2014). The physiological substrates of neddylation are cullin family members, which are the scaffold subunit of cullin-RING ligase (CRL), whose activity requires cullin neddylation (Zhao et al., 2014). The founding member of CRL is SCF (SKP1, Cullin-1, and F-box protein) E3 ubiquitin ligase, which promotes ubiquitylation and degradation of many short-lived signal molecules (Nakayama and Nakayama, 2006). In our effort to elucidate potential transcription regulation of MLN4924, we performed an RNAseq analysis and found that SOX2 transcription is significantly down-regulated by MLN4924 in time and dose dependent manner. Mechanistic study revealed that MLN4924 caused accumulation of MSX2, a transcription repressor known to suppress SOX2 expression (Wu et al., 2015). Further investigation identified MSX2 as a substrate of SCF^FBXW2^ E3 ligase for targeted ubiquitylation and degradation. By inhibiting Cullin-1 neddylation, MLN4924 inactivates SCF^FBXW2^ E3 ligase to cause accumulation of MSX2, leading to SOX2 downregulation. Biologically, FBXW2 promotes tumor sphere formation by upreglating SOX2 via inducing MSX2 degradation, while MLN4924 sensitizes breast cancer cells to tamoxifen by depleting SOX2 via targeting the FBXW2-MSX2 axis (Yin et al., 2019). Our study, therefore, provides a novel mechanism by which MLN4924 regulates cancer stem cell property and overcomes tamoxifen resistance with potential therapeutic application in targeting human cancers with SOX2 overexpression.

**Figure 1:**
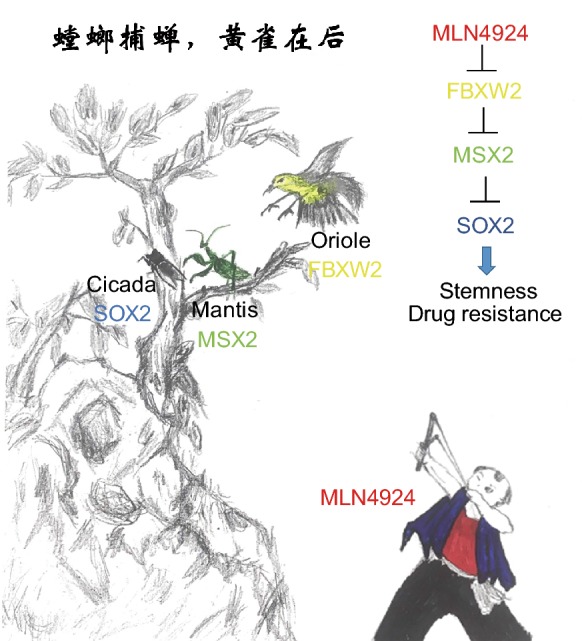
**A negative cascade of the FBXW2–MSX2–SOX2 axis is targeted by MLN4924**. SOX2 (cicada) is a transcriptional downstream target of MSX2 (mantis). Upon binding to SOX2 gene promoter, MSX2 negatively controls SOX2 transcription. MSX2, on the other hand, is a substrate of FBXW2 (oriole) E3 ligase. Upon hypoxia, FBXW2 binds to MSX2 and targets it for ubiquitylation and subsequent degradation. By inactivating FBXW2, MLN4924 (man with a slingshot) causes MSX2 accumulation to transcriptionally repress SOX2

Our study can extend an old Chinese saying: “The mantis stalks the cicada, unaware of the oriole lurking behind itself (**螳螂捕蝉黄雀在后)**” by adding “and oriole is unware of a man with slingshot behind itself (Fig. 1). Here we designate the MLN4924 to be a man with slingshot, who ultimately targets the cicada (SOX2) via oriole (FBXW2) and mantis (MSX2), to regulate stem cell property and sensitize breast cancer cells to tamoxifen.
